# Not All Worms Were Created Equal

**DOI:** 10.3389/fimmu.2022.877707

**Published:** 2022-03-10

**Authors:** Alex Loukas, John Croese, Megan A. Rees, James S. McCarthy

**Affiliations:** ^1^ Australian Institute of Tropical Health and Medicine, James Cook University, Cairns, QLD, Australia; ^2^ Department of Respiratory Medicine and Sleep Disorders, Royal Melbourne Hospital, Melbourne, VIC, Australia; ^3^ Victorian Infectious Diseases Service, Royal Melbourne Hospital, and Peter Doherty Institute of Infection and Immunity, Melbourne, VIC, Australia

**Keywords:** hookworm, helminth therapy, lungs, *Necator americanus*, respiratory

Whilst we support the need to report safety outcomes from experimental therapy of any kind, including the use of helminths as therapy, we believe that it is important to critically examine the causal relationship of the reported event to the proposed etiology so that a balanced view of the cause and effect be arrived at. This is particularly the case in an uncontrolled setting where formal processes for documenting and reporting experimental therapy may not be in place.

Recent reviews of studies with one of the most widely used helminths, the anthropophilic hookworm, *Necator americanus*, have shown this helminth to be safe and well tolerated in hundreds of individuals by numerous research groups across Australia, the US and Europe (1–3). In BMC Pulmonary Medicine (4), Zeynalyan and colleagues report rapidly progressive respiratory failure in a patient with significant comorbidities, including systemic sclerosis, interstitial lung disease and pulmonary hypertension after self-administration of *N. americanus* larvae that were purchased over the internet. Here we raise some concerns about this report.

The life cycle, tissue tropism, migratory path and pathogenic sequelae differ widely between helminths, and even between closely related species. For example, the human hookworm *Necator americanus*, the species typically used in human infection studies is very poorly infectious when applied orally (0.8%) as was the case in this report (4), compared to the closely related human hookworm *Ancylostoma duodenale* (50% infective *via* oral route) (3). Secondly, although Loeffler’s syndrome is a well described complication of *Ascaris* infection, it should not be confused with the distinct respiratory features associated with hookworm infection. In a detailed account of respiratory symptoms following dermal inoculation with a large dose (400 larvae) of *A. duodenale*, Brumpt reported retro-sternal chest pain, dry hacking, non-productive cough and dysphonia, with a nocturnal predominance which commenced from the fourth day after inoculation, and persisted for up to 3 weeks (5). However, on physical examination, signs of pulmonary consolidation were absent and no chest X-ray changes were reported. Likewise, Lee et al. reported transient respiratory symptoms from days 7 to 21 in subjects inoculated with a mix of 150-800 *A. duodenale* and *Ancylostoma ceylanicum* larvae (6). Symptoms correlate with the ascent of larvae into the trachea, at which point larvae may be isolated from sputa (7). Unlike Loeffler’s syndrome, eosinophilia is not present during pulmonary migration of hookworms but only becomes apparent when the parasites reach the intestine. In contrast, in the case reported by Zeynalyan et al., the respiratory symptoms began six weeks after the last ingestion of larvae (4). Further, the rise in eosinophil count would be consistent with timing of parasites reaching the small intestine, when eosinophilia typically develops, and was much lower than typically reported with Loeffler’s syndrome.

In the case reported by Zeynalyan and colleagues the definitive diagnostic test for infection (helminth eggs in the feces) was negative, while *Strongyloides* serology was positive (4). *Strongyloides* serodiagnosis has been shown to not cross-react with hookworm infected subjects (8).

Finally, the clinical course for patients with advanced interstitial lung disease due to systemic sclerosis and pulmonary hypertension is frequently noted for a progressive deterioration with a shortened life expectancy (9).

We believe that the case reported by Zeynalyan has merit in reporting the risks of unregulated experimentation outside the bounds of a carefully conducted clinical trial, and highlights the importance of using appropriately regulated products in clinical trials.

*N. americanus* therapy has been, and continues to be methodically and rigorously tested in clinical environments where the source of the infective inocula is meticulously monitored and validated (1, 3, 10). Purchasing helminths on the internet and administering them without careful clinical oversight should be discouraged. Publication of adverse outcomes of poorly controlled clinical studies should highlight the specific risks of this aspect rather than suggest danger from the ethical conduct of clinical trials to test a well-defined hypothesis in a controlled setting.

## Author Contributions

AL, JC and JM led the writing of the opinion article. All authors contributed to the article and approved the submitted version.

## Conflict of Interest

The authors declare that the research was conducted in the absence of any commercial or financial relationships that could be construed as a potential conflict of interest.

## Publisher’s Note

All claims expressed in this article are solely those of the authors and do not necessarily represent those of their affiliated organizations, or those of the publisher, the editors and the reviewers. Any product that may be evaluated in this article, or claim that may be made by its manufacturer, is not guaranteed or endorsed by the publisher.
